# Supplementary material for: PBRM1 loss defines a nonimmunogenic tumor phenotype associated with checkpoint inhibitor resistance in renal carcinoma
Source: Nat Commun. 2020 May 1;11:2135. doi: 10.1038/s41467-020-15959-6 (PMC7195420; doi:10.1038/s41467-020-15959-6)
Supplement: Supplementary file 1 — Supplementary Information [file 41467_2020_15959_MOESM1_ESM.pdf]

PBRM1 loss defines a nonimmunogenic tumor phenotype associated with checkpoint inhibitor resistance in renal carcinoma

Liu et al.

Supplementary Information

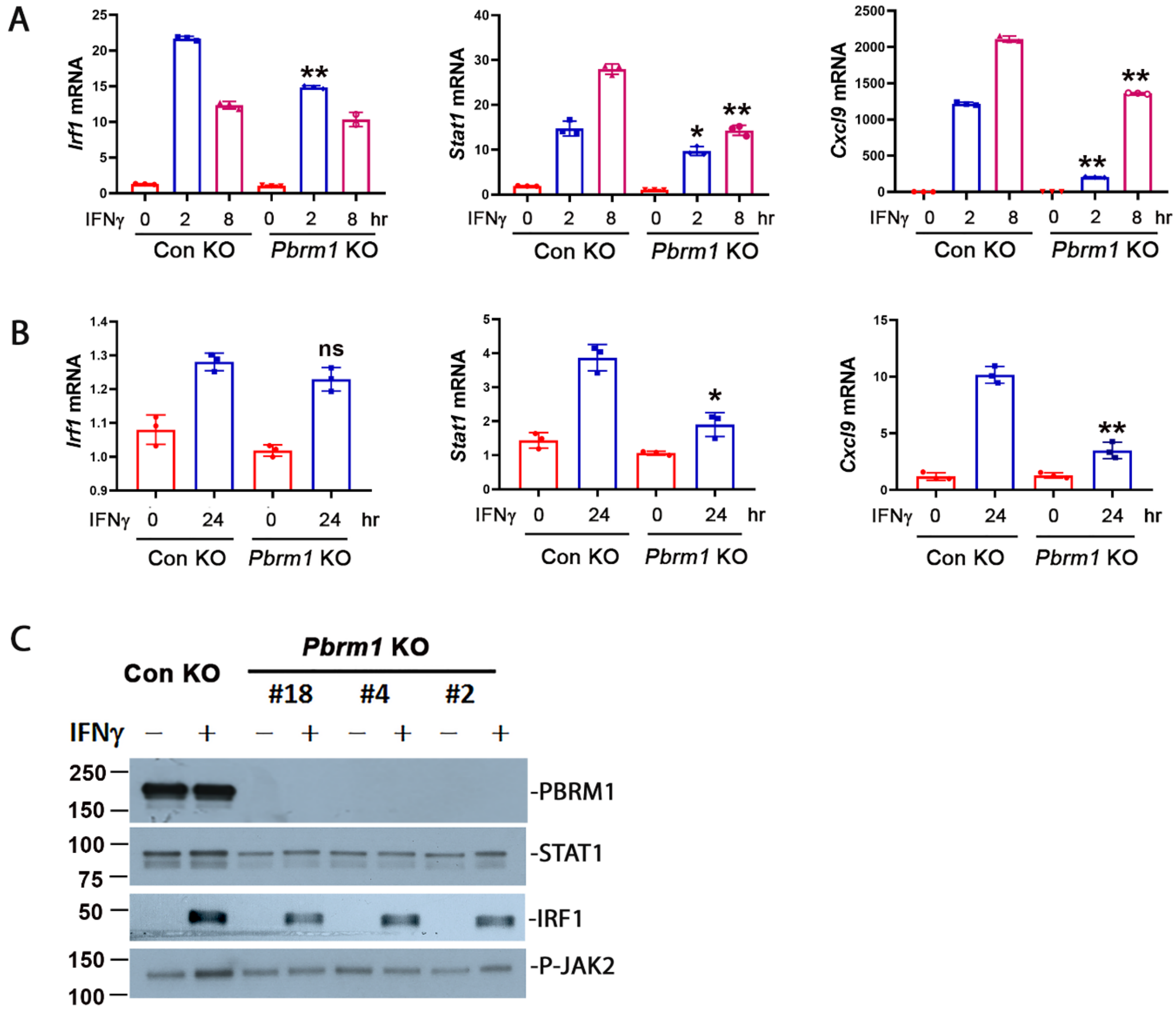

Supplementary Figure 1. PBRM1 loss reduced IFNγ-induced gene expression. (A-B) IFNγ-induced gene expression. mRNA expression of *Irf1, Stat1* and *Cxcl9* were detected by real-time PCR. *Gapdh* was used as internal control. (C) IFNγ-induced expression of STAT1, IRF1 and JAK2 phosphorylation. Cell lysates were analyzed by immunoblot using antibodies against PBRM1, STAT1, P-JAK2 Y1007/1008 and IRF1. Renca cells were cultured in DMEM with 10% FBS, and treated with 1ng/ml IFNγ for indicated number of hours. Data in the bar graphs represent mean±S.D., n=3. Unpaired t-test was performed with GraphPad Prism 7.03. ns, not significant, *, *P*<0.05 and **, *P*<0.001, compared with control knockout cells. All data are representative of three independent experiments. Source data are provided as a Source Data file.

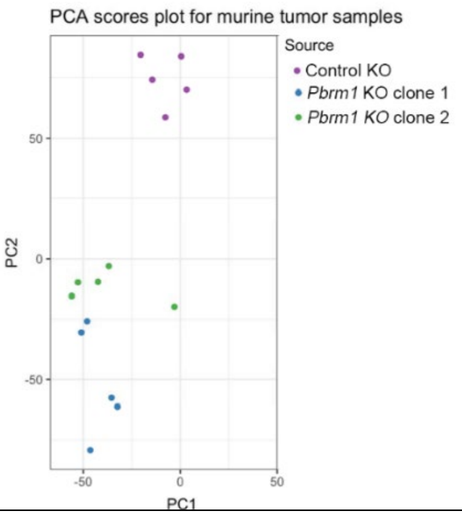

Supplementary Figure 2. Principal Component Analysis (PCA) on the full set of 18279 genes in both control and *Pbrm1* knockout tumors to detect the overall variation in the data and to check for outliers.

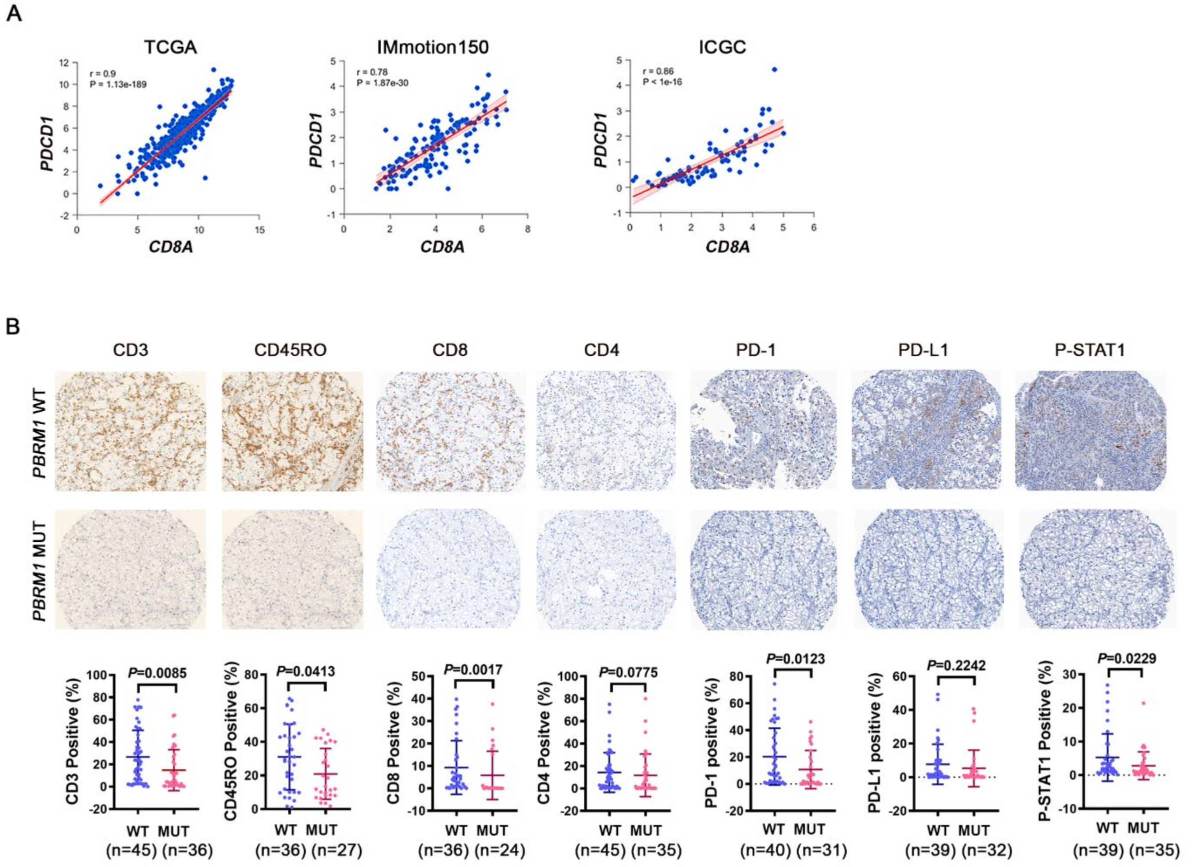

Supplementary Figure 3. PBRM1 loss was associated with less T cell infiltration in sunitinib-treated RCC samples. (A) Correlation between expression levels of *CD8A* and *PDCD1* (*PD-1*) in ccRCC patients from TCGA (N = 440), IMmotion150 (N = 134), and ICGC (N = 118). Inset value indicates Spearman correlation coefficient and corresponding P-value. (B) T cell infiltration and quantification. RCC tissue microarrays from sunitinib-treated RCC patients were immunohistochemically stained with antibodies against CD3, CD45RO, CD8, CD4, PD-1, PD-L1 and P-STAT1 Y701. The percentages of positively stained cells were analyzed using inForm software. Non-parametric Mann-Whitney test was performed with GraphPad Prism 7.03. Source data are provided as a Source Data file.

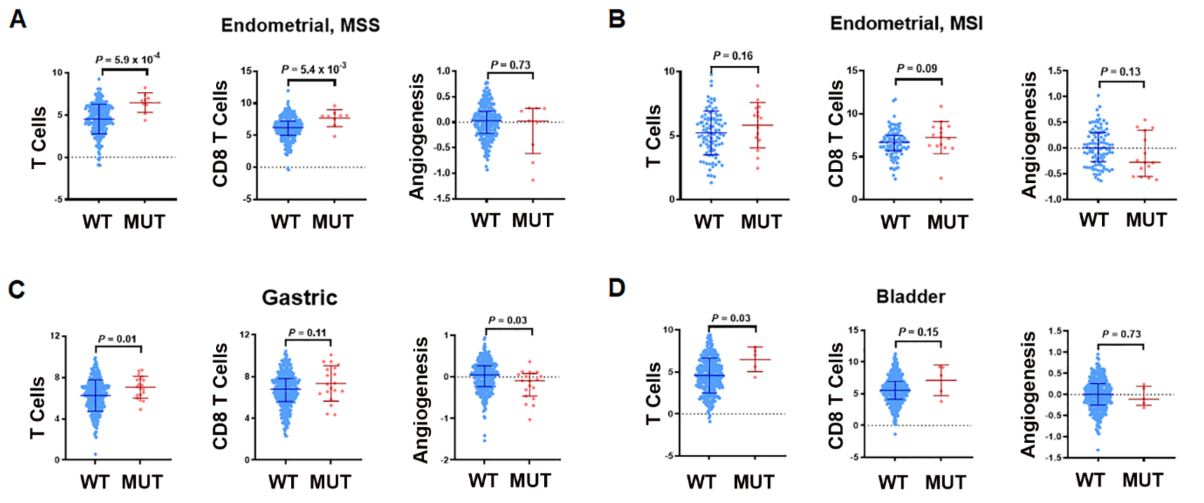

Supplementary Figure 4. Effect of PBRM1 loss on immune infiltration and angiogenesis in other cancer lineages. Gene expression-based inference of total T cell and CD8 T cell infiltrates in patients stratified by *PBRM1* mutation status, as well as angiogenesis score shown for TCGA tumor samples from (A) microsatellite stable (MSS) endometrial cancer cohort, (B) microsatellite instable (MSI) endometrial cancer cohort, (C) gastric cancer cohort, and (D) bladder cancer cohort. Rank-sum test. Source data are provided as a Source Data file.

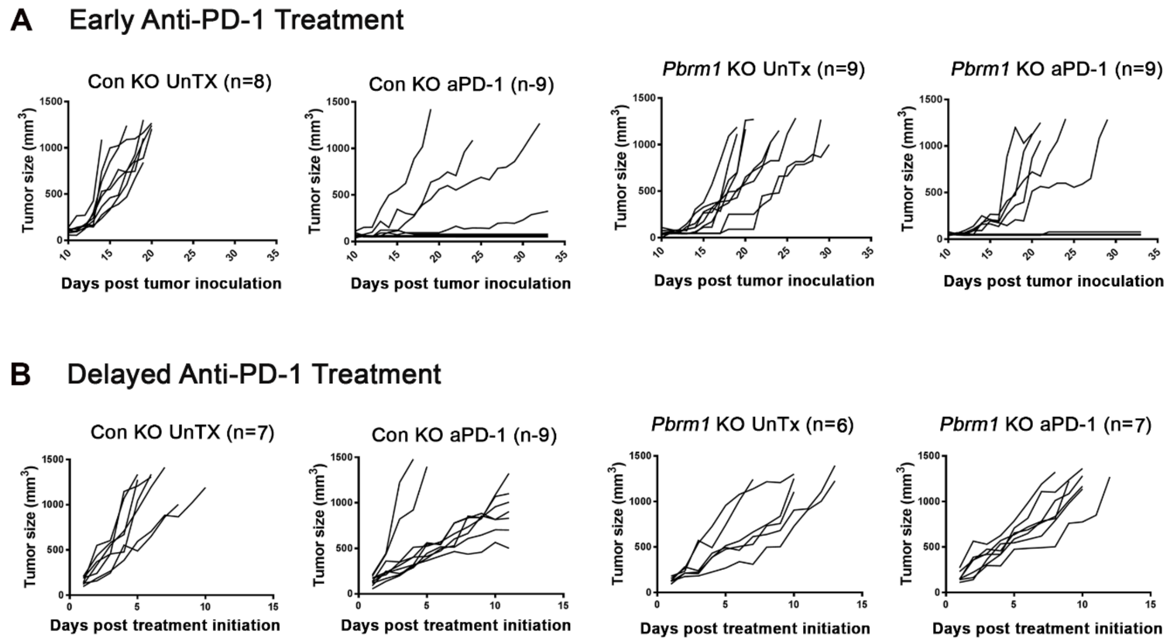

Supplementary Figure 5. PBRM1 loss induced resistance to ICB. A. *In vivo* tumor growth rates in Renca control KO and *Pbrm1* KO tumors with or without early anti-PD-1 treatments. PD-1 antibodies were administrated at day 3, day 6 and day 9 after tumor inoculation. First dose was 400μg/mouse, and the following two doses were 200 μg/mouse. B. *In vivo* tumor growth rates in Renca control KO and *Pbrm1* KO tumors with or without delayed anti-PD-1 treatment. Anti-PD-1 antibody (200 μg/mouse) was administrated every third day once the tumors reached 100-200 mm^3. UnTX, untreated control. Source data are provided as a Source Data file.

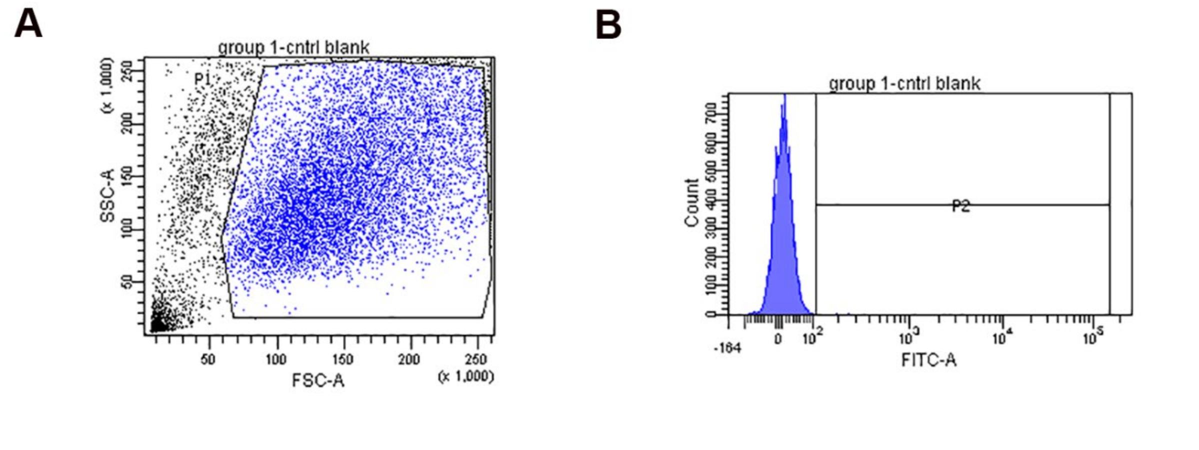

Supplementary Figure 6. Gating strategy. For all flow cytometry analysis, live, healthy cells were gated by FSC and SSC to remove dead cells with high SSC and debris in the lower left quadrant of dot plot. Unstained negative control cells were used to establish the boundary of the negative signal in the FITC channel.

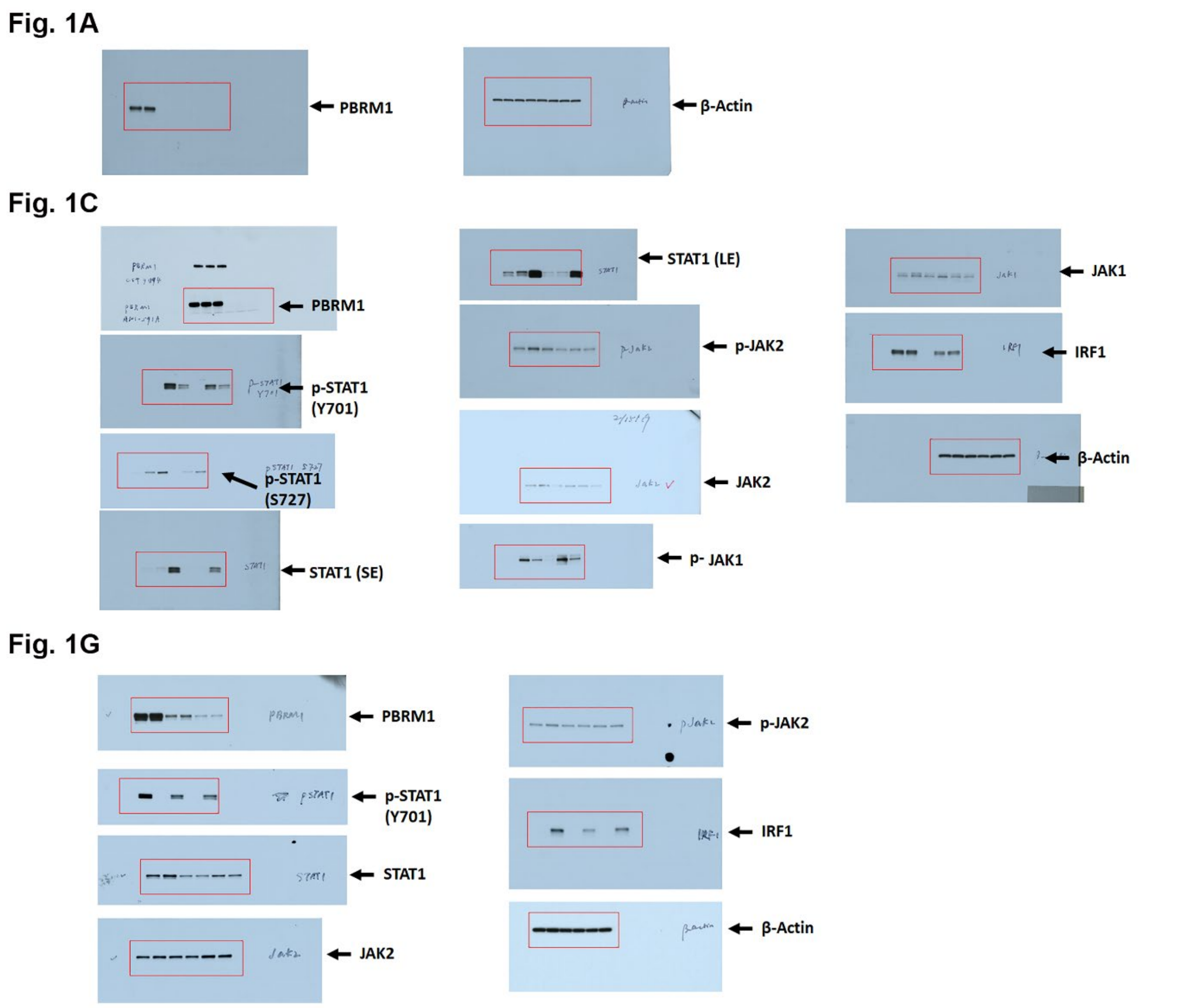

Supplementary Figure 7. Western blot results of the influence of PBRM1 loss on IFNγ-STAT1 signaling (Fig.1A, C, G).

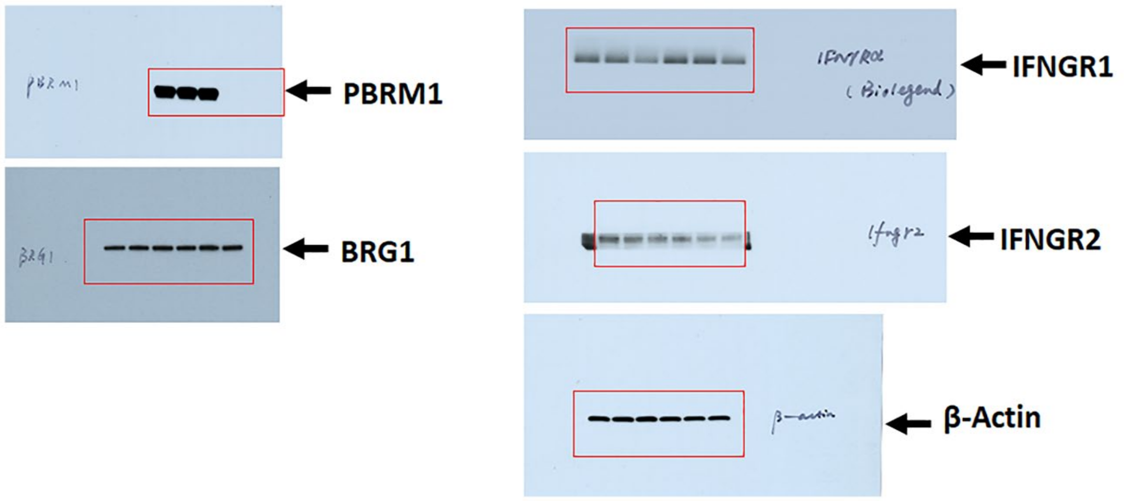

Supplementary Figure 8. Western blot results of the influence of Pbrm1 knockout on IFNGR2 expression (Fig.2C).

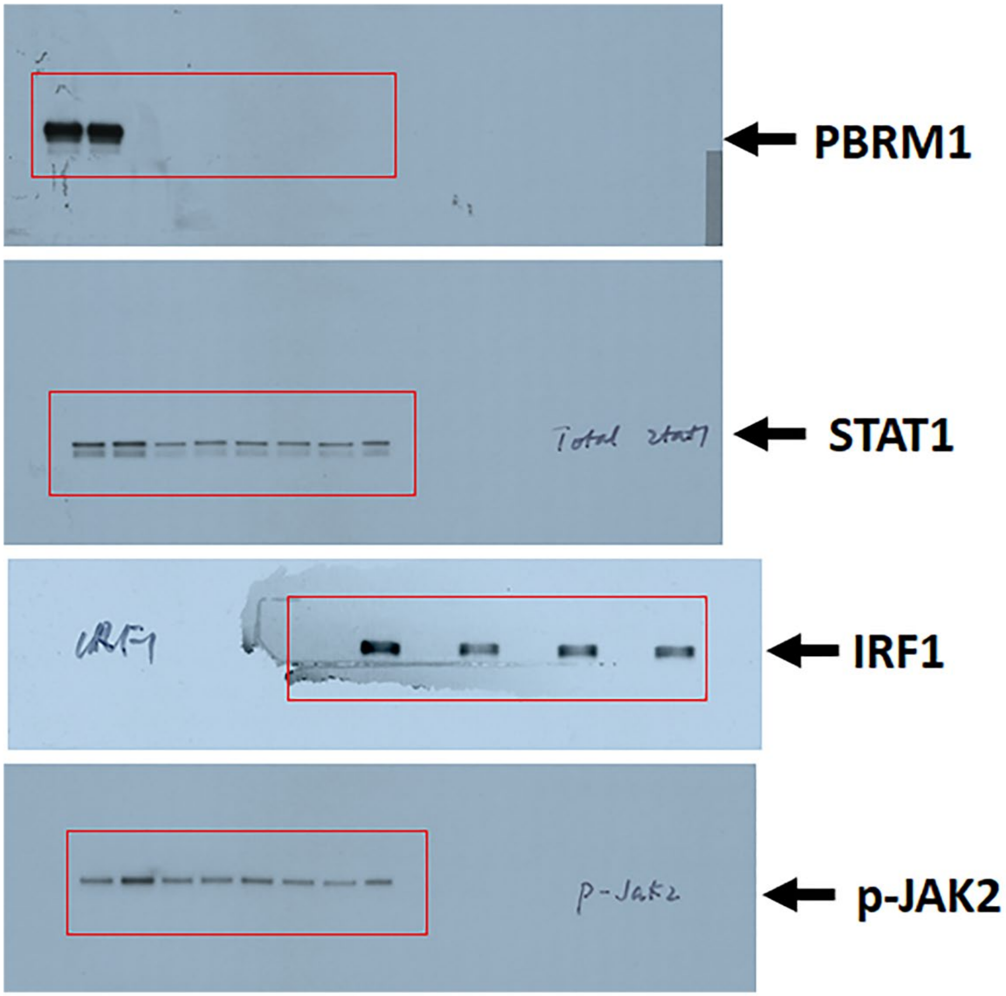

Supplementary Figure 9. Western blot results of the influence of *Pbrm1* KO on IFNγ-STAT1 signaling (Fig.S1C).

Supplementary Table. 1

Real Time PCR Primers for Gene Expression:

Murine Genes	Forward primer	Reverse Primer
Pbrm1	5'-TGACAGGTCCTTCGCACAATA-3'	5'-TCTGATCCATACTGAAGTGCCA-3'
Stat1	5'-GCTGCCTATGATGTCTCGTTT-3'	5'-TGCTTTTCCGTATGTTGTGCT-3'
Irf1	5'-ATGCCAATCACTCGAATGCG-3'	5'-TTGTATCGGCCTGTGTGAATG-3'
Ifng	5'-ACAGCAAGGCGAAAAAGGATG-3'	5'-TGGTGGACCACTCGGATGA-3'
Pdcd1	5'-ACCCTGGTCATTCACTTGGG-3'	5'-CATTTGCTCCCTCTGACACTG-3'
Cd274	5'-AGTATGGCAGCAACGTCACG-3'	5'-TCCTTTTCCCAGTACACCACTA-3'
Cxcl9 [1]	5'-AGTCCGCTGTTCTTTTCCTC-3'	5'-TGAGGTCTTTGAGGGATTTGTAG-3'
Cxcl10	5'-CCAAGTGCTGCCGTCATTTTC-3'	5'-GGCTCGCAGGGATGATTTCAA-3'
Ciita	5'-GGAGGAGATCGAACTCAGCTC-3'	5'-GTTCCGCAATGTTGGCATAGG-3'
H2-Aa	5'-TCAGTCGCAGACGGTGTTTAT-3'	5'-GGGGGCTGGAATCTCAGGT-3'
Icam1	5'-TGCCTCTGAAGCTCGGATATAC-3'	5'-TCTGTCGAACTCCTCAGTCAC-3'
Ifngr1	5'-CTGGCAGGATGATTCTGCTGG-3'	5'-GCATACGACAGGGTTCAAGTTAT
Ifngr2	5'-TCCTCGCCAGACTCGTTTTC-3'	5'-GTCTTGGGTCATTGCTGGAAG-3'
Gapdh	5'-AGGTCGGTGTGAACGGATTTG-3'	5'GGGGTCGTTGATGGCAACA-3'
Human Genes	Forward primer	Reverse Primer
PBRM1	5'-AGGAGGAGACTTTCCAATCTTCC-3'	5'- CTTCGCTTTGGTGCCCTAATG-3'
STAT1	5'-ATCAGGCTCAGTCGGGGAATA-3'	5'-TGGTCTCGTGTTCTCTGTTCT-3'
STAT2	5'-GAGCCAGCAACATGAGATTGA-3'	5'-GCCTGGATCTTATATCGGAAGCA-3'
CXCL9	5'-CCAGTAGTGAGAAAGGGTCGC-3'	5'-AGGGCTTGGGGCAAATTGTT-3'
IRF1	5'-CTGTGCGAGTGTACCGGATG-3'	5'-ATCCCCACATGACTTCCTCTT-3'
IRF9	5'-GCCCTACAAGGTGTATCAGTTG-3'	5'-TGCTGTCGCTTTGATGGTACT-3'
IFNGR1	5'-TCTTTGGGTCAGAGTTAAAGCCA-3'	5'-TTCCATCTCGGCATACAGCAA-3'
IFNGR2	5'-CTCCTCAGCACCCGAAGATTC-3'	5'-GCCGTGAACCATTTACTGTCG-3'
GAPDH	5'-CAATGACCCCTTCATTGACC-3'	5'-TTGATTTTGGAGGGATCTCG-3'

Real Time PCR Primers for ChIP:

Genes	Forward primer	Reverse Primer
Cxcl9 [2]	5'-TTCCACATCCAGGTAGCAACTTTG-3'	5'-TGTTGGAGTGAAGTCCGAGAATGT-3'
Cxcl10 [3]	5'-CCTGTAAACCGAGGGCATTG-3'	5'- CACGCTTTGGAAAGTGAAAC-3'
Ifngr2 [4]	5'- GGGGTACAGATCCAGGGAAT-3'	5'- CAGCTAAAGCCACGAAGGAC-3'

Supplementary Table. 1 Primers used for real time PCR. All the sequences were from PrimerBank (https://pga.mgh.harvard.edu/primerbank/) except those with indicated references.

References:

1. Pan, D., *et al.* A major chromatin regulator determines resistance of tumor cells to T cell-mediated killing. *Science* **359**, 770-775 (2018).
2. Hiroi, M. & Ohmori, Y. The transcriptional coactivator CREB-binding protein cooperates with STAT1 and NF-kappa B for synergistic transcriptional activation of the CXC ligand 9/monokine induced by interferon-gamma gene. *J Biol Chem* **278**, 651-660 (2003).
3. Chmielewski, S., *et al.* STAT1-dependent signal integration between IFNgamma and TLR4 in vascular cells reflect pro-atherogenic responses in human atherosclerosis. *PLoS One* **9**, e113318 (2014).
4. Huang, N., Tan, L., Xue, Z., Cang, J. & Wang, H. Reduction of DNA hydroxymethylation in the mouse kidney insulted by ischemia reperfusion. *Biochem Biophys Res Commun* **422**, 697-702 (2012).
