# Supplementary material for: PBRM1 loss defines a nonimmunogenic tumor phenotype associated with checkpoint inhibitor resistance in renal carcinoma
Source: Nat Commun. 2020 May 1;11:2135. doi: 10.1038/s41467-020-15959-6 (PMC7195420; doi:10.1038/s41467-020-15959-6)
Supplement: Supplementary file 3 — Reporting Summary [file 41467_2020_15959_MOESM3_ESM.pdf]

nature research

Corresponding author(s): Xian-De Liu; Eric Jonasch

Last updated by author(s): 03-18-2020

Reporting Summary

Nature Research wishes to improve the reproducibility of the work that we publish. This form provides structure for consistency and transparency in reporting. For further information on Nature Research policies, see Authors & Referees and the Editorial Policy Checklist .

Statistics

For all statistical analyses, confirm that the following items are present in the figure legend, table legend, main text, or Methods section.

n/a	Confirmed	
☐	☒	The exact sample size (*n*) for each experimental group/condition, given as a discrete number and unit of measurement
☐	☒	A statement on whether measurements were taken from distinct samples or whether the same sample was measured repeatedly
☐	☒	The statistical test(s) used AND whether they are one- or two-sided<br>*Only common tests should be described solely by name; describe more complex techniques in the Methods section.*
☐	☒	A description of all covariates tested
☒	☐	A description of any assumptions or corrections, such as tests of normality and adjustment for multiple comparisons
☐	☒	A full description of the statistical parameters including central tendency (e.g. means) or other basic estimates (e.g. regression coefficient) AND variation (e.g. standard deviation) or associated estimates of uncertainty (e.g. confidence intervals)
☒	☐	For null hypothesis testing, the test statistic (e.g. *F*, *t*, *r*) with confidence intervals, effect sizes, degrees of freedom and *P* value noted<br>*Give P values as exact values whenever suitable.*
☒	☐	For Bayesian analysis, information on the choice of priors and Markov chain Monte Carlo settings
☒	☐	For hierarchical and complex designs, identification of the appropriate level for tests and full reporting of outcomes
☒	☐	Estimates of effect sizes (e.g. Cohen's *d*, Pearson's *r*), indicating how they were calculated

Our web collection on statistics for biologists contains articles on many of the points above.

Software and code

Policy information about availability of computer code

Data collection	Patient data from TCGA is available from the TCGA data portal (https://portal.gdc.cancer.gov/). Patient data from ICGC is available through the ICGC data portal (https://dcc.icgc.org/). Data from the IMmotion150 trial were downloaded from European Genome-Phenome Archive (EGA) under accession number EGAS00001002928. Data for pre-malignant murine kidneys were acquired from GEO accession GSE83597. Data for Renca tumors were deposited to The Gene Expression Omnibus (GEO) (GSE145919, https://www.ncbi.nlm.nih.gov/geo/query/acc.cgi?acc=GSE145919).
Data analysis	Expression data for TCGA patients either was imported into R using the TCGA2STAT package 62 or imported manually into Matlab (2016b). Mouse sequencing reads were aligned to mouse reference genome (mm10) with tophat2, and the gene-based read counts were generated by HTSeq, then the raw counts were normalized with R package DESeq 63. RNAseq data from the IMMotion150 trial was quantified using kallisto (v0.44.0) 66. GSEA was run using the software provided by the Broad Institute at http://software.broadinstitute.org/gsea/index.jsp. Any applicable custom scripts are available from the authors upon request.

For manuscripts utilizing custom algorithms or software that are central to the research but not yet described in published literature, software must be made available to editors/reviewers. We strongly encourage code deposition in a community repository (e.g. GitHub). See the Nature Research guidelines for submitting code & software for further information.

Data

Policy information about availability of data

All manuscripts must include a data availability statement. This statement should provide the following information, where applicable:

- Accession codes, unique identifiers, or web links for publicly available datasets
- A list of figures that have associated raw data
- A description of any restrictions on data availability

Statistical source data for graphical representations and statistical analysis in Figs. 1(B, D, E, G), 2(A-C), 3(B,C,E,G,H), 4 (E,G), 5 (A,D-F), 6 (A,B,E,F), and supplementary Figs S1 (A,B), S3(B), S4 (A-D) and S5 (A,B) are provided in PBRM1-immunigenicity-SourceData file. Uncropped western blot images are available in Supplementary Fig. 6-8. Patient data from TCGA is available from the TCGA data portal (https://portal.gdc.cancer.gov/). Patient data from ICGC is available through

the ICGC data portal (https://dcc.icgc.org/). Data from the IMmotion150 trial were downloaded from European Genome-Phenome Archive (EGA) under accession number EGAS00001002928. Data for pre-malignant murine kidneys were acquired from GEO accession GSE83597. Data for Renca tumors were deposited to The Gene Expression Omnibus (GEO) (GSE145919, https://www.ncbi.nlm.nih.gov/geo/query/acc.cgi?acc=GSE145919). For patients from the MSKCC IMPACT study, survival data for ICB-treated patients was acquired from Samstein et. al. 48, and mutation data was downloaded from cBioPortal (https://www.cbioportal.org/) 67. All other data that support the findings of this study are available from the corresponding author upon reasonable request.

Field-specific reporting

Please select the one below that is the best fit for your research. If you are not sure, read the appropriate sections before making your selection.

[x] Life sciences [ ] Behavioural & social sciences [ ] Ecological, evolutionary & environmental sciences

For a reference copy of the document with all sections, see nature.com/documents/nr-reporting-summary-flat.pdf

Life sciences study design

All studies must disclose on these points even when the disclosure is negative.

Sample size	The minimal sample size is at least 3, and student t test was used for statistical predetermine.
Data exclusions	No data were excluded
Replication	All attempts at replication were successful.
Randomization	Mice bearing tumors were randomly allocated into experimental groups.
Blinding	Our lab tech, Xuesong Zhang, were blinded for data collection for animal experiments.

Reporting for specific materials, systems and methods

We require information from authors about some types of materials, experimental systems and methods used in many studies. Here, indicate whether each material, system or method listed is relevant to your study. If you are not sure if a list item applies to your research, read the appropriate section before selecting a response.

Materials & experimental systems

n/a	Involved in the study	
[ ]	[x]	Antibodies
[ ]	[x]	Eukaryotic cell lines
[x]	[ ]	Palaeontology
[ ]	[x]	Animals and other organisms
[x]	[ ]	Human research participants
[x]	[ ]	Clinical data

Methods

n/a	Involved in the study	
[x]	[ ]	ChIP-seq
[ ]	[x]	Flow cytometry
[x]	[ ]	MRI-based neuroimaging

Antibodies

Antibodies used	PBRM1 antibody (A301-591A) was from Bethyl Laboratories. Phospho-STAT1 antibody (clone ST1P-11A5; Tyr701; 33-3400), human CD3 antibody (clone F7.2.38; MA5-12577), human CD45RO antibody (clone UCHL1; MA5-11532), human CD4 antibody (clone 4B12; MS1528S0) and human CD8 antibody (clone C8/144B; MS457S0) were from ThermoFisher Scientific. Mouse CD3 antibody (D4V8L; 99940), mouse CD8 antibody(D4W22; 98941), mouse CD4 antibody (D7D2Z; 25229), human PD-L1 antibody (E1L3N; 13684), mouse PD-L1 antibody (D5V3B; 64988), mouse PD-1 antibody (D7D5W; 84651), PBRM1 antibody (D3F7O, 91894), JAK1 antibody (6G4, 3344), JAK2 antibody (D2E12, 3230), Phospho-JAK1 antibody (D7N4Z, Tyr1034/1035, 74129), Phospho-JAK2 antibody (C80C3, Tyr1007/1008, 3776), STAT1 antibody (D1K9Y, 14994), Phospho-STAT1 antibody (58D6, Tyr701, 9167), Phospho-STAT1 antibody (D3B7, Ser727, 8826), IRF1 antibody (D5E4, 8478) and BRG1 antibody (E9O6E; 52251)were from cell signaling technology. IFNGR1 antibody (112802) was from Biolegend. IFNGR2 antibody (Cat No. GTX 64548) was from GeneTex. β-actin antibody (A1978) was from Sigma.
Validation	All of the antibodies we bought were described as validated on manufacture`s website by attached related results (Western blot, ChIP or flow cytometry). In addition, we also further validate the specificity in our experiments based on molecular weight, reduced expression by knockout/knock down, or inducted expression upon stimulation.

Eukaryotic cell lines

Policy information about cell lines

Cell line source(s)	Renca cell line and 786-O cell line were from ATCC.
Authentication	786-O cell line were obtained from the ATCC. All cell lines were validated by short tandem repeat (STR) DNA fingerprinting using the Promega 16 High Sensitivity STR Kit.
Mycoplasma contamination	All cell lines were tested as mycoplasma negative
Commonly misidentified lines (See ICLAC register)	*Name any commonly misidentified cell lines used in the study and provide a rationale for their use.*

Animals and other organisms

Policy information about studies involving animals; ARRIVE guidelines recommended for reporting animal research

Laboratory animals	Four to six week-old-female BALB/c mice were purchased from TACONIC.
Wild animals	*Provide details on animals observed in or captured in the field; report species, sex and age where possible. Describe how animals were caught and transported and what happened to captive animals after the study (if killed, explain why and describe method; if released, say where and when) OR state that the study did not involve wild animals.*
Field-collected samples	*For laboratory work with field-collected samples, describe all relevant parameters such as housing, maintenance, temperature, photoperiod and end-of-experiment protocol OR state that the study did not involve samples collected from the field.*
Ethics oversight	The animal protocols were approved by Institutional Animal Care and Use Committee (IACUC) of The Health Science Center, Texas A&M University.

Note that full information on the approval of the study protocol must also be provided in the manuscript.

Flow Cytometry

Plots

Confirm that:

- [x] The axis labels state the marker and fluorochrome used (e.g. CD4-FITC).
- [x] The axis scales are clearly visible. Include numbers along axes only for bottom left plot of group (a 'group' is an analysis of identical markers).
- [x] All plots are contour plots with outliers or pseudocolor plots.
- [x] A numerical value for number of cells or percentage (with statistics) is provided.

Methodology

Sample preparation	1. Harvest 1 × 10^6 cells per sample in a 15 mL tube, wash with cold 1X PBS + 2% FBS<br>2. Spin 5 min. at 300g, 4˚C , and then aspirate<br>3. Gently resuspend with 100 µL PBS + 2% FBS<br>4. Divide volume evenly into two 5 mL flow tubes (50 µL per tube, one tube for control without antibody, and one for specific Ab-labeled samples)<br>5. Add IFNGR2-FITC antibody to appropriate tubes and incubate on ice for 20 min in the dark (cover ice bucket with aluminum foil)<br>6. Wash with 3 mL cold PBS + 2% FBS, Spin 5 min at 300g, 4˚C and aspirate<br>7. Fix with 400 µL 4% Formaldehyde on ice for 15 min.<br>8. Store in the dark at 4˚C and analyze samples on flow cytometer
Instrument	BD LSR II
Software	FACSDiva Version 6.2
Cell population abundance	Cell sorting was not required for our experiment. When we generate knockout cell lines, we picked up single clones, and the cell populations were 100% pure, as indicated by complete PBRM1 knockout in Fig. 1A.

Gating strategy

For all flow cytometry analysis, live, healthy cells were gated by FSC and SSC to remove dead cells with high SSC and debris in the lower left quadrant of dot plot. Unstained negative control cells were used to establish the boundary of the negative signal in the FITC channel. For the Ab labeled samples, any fluorescent events to the right of the negative gate were considered positive and measured on a percentage basis.

☒ Tick this box to confirm that a figure exemplifying the gating strategy is provided in the Supplementary Information.
